# Energy Stores, Oxidative Balance, and Sleep in Migratory Garden Warblers (*Sylvia borin*) and Whitethroats (*Sylvia communis*) at a Spring Stopover Site

**DOI:** 10.1093/iob/obaa010

**Published:** 2020-04-15

**Authors:** Andrea Ferretti, Scott R McWilliams, Niels C Rattenborg, Ivan Maggini, Massimiliano Cardinale, Leonida Fusani

**Affiliations:** 1 Department of Behavioural and Cognitive Biology, University of Vienna, Althanstraße 14 (UZA1), Wien 1090, Austria; 2 Konrad Lorenz Institute of Ethology, University of Veterinary Medicine, Vienna, Savoyenstraße 1a, Wien 1160, Austria; 3 Department of Natural Resources Science, University of Rhode Island, 1 Greenhouse Road, Kingston, RI 02881, USA; 4 Avian Sleep Group, Max Planck Institute for Ornithology, Eberhard-Gwinner-Straße, Seewiesen 8231, Germany; 5 Marine Research Institute, Swedish University of Agricultural Sciences, Turistgatan 5, Lysekil SE-453 30, Sweden

## Abstract

Little is known about how songbirds modulate sleep during migratory periods. Due to the alternation of nocturnal endurance flights and diurnal refueling stopovers, sleep is likely to be a major constraint for many migratory passerine species. Sleep may help to increase the endogenous antioxidant capacity that counteracts free radicals produced during endurance flight and reduces energy expenditure. Here, we investigated the relationship between sleep behavior, food intake, and two markers of physiological condition—the amount of energy reserves and oxidative status—in two migratory songbird species, the garden warbler (*Sylvia borin*) and the whitethroat (*Sylvia communis*). In garden warblers, birds with high energy stores were more prone to sleep during the day, while this condition-dependent sleep pattern was not present in whitethroats. In both species, birds with low energy stores were more likely to sleep with their head tucked in the feathers during nocturnal sleep. Moreover, we found a positive correlation between food intake and the extent of energy reserves in garden warblers, but not in whitethroats. Finally, we did not find significant correlations between oxidative status and sleep, or oxidative status and energy stores. Despite our study was not comparative, it suggests that different species might use different strategies to manage their energy during stopover and, additionally, it raises the possibility that migrants have evolved physiological adaptations to deal with oxidative damage produced during migration.

## Synopsis (ITA)

Il pattern del sonno nei passeriformi migratori è praticamente sconosciuto. Durante la migrazione, il sonno può diventare una importante limitazione fisiologica per molte specie che alternano estenuanti voli notturni con intensi foraggiamenti diurni per recuperare le energie. Oltre a ridurre il dispendio energetico, il sonno potrebbe promuovere le capacità antiossidanti endogene che contrastano i radicali liberi che si accumulano durante i voli prolungati. In questo studio, abbiamo indagato la relazione tra sonno, assunzione di cibo, e due marcatori della condizione fisiologica—la quantità di riserve di energia e lo stato ossidativo—in due specie di passeriformi migratori, il Beccafico (*Sylvia borin*) e la Sterpazzola (*Sylvia communis*). Nei Beccafichi, gli individui con abbondanti riserve energetiche erano più inclini a dormire durante il giorno che durante la notte, mentre non è stata trovata una associazione significativa tra il pattern di sonno e le riserve energetiche nelle Sterpazzole. In entrambe le specie, gli uccelli con scarse riserve energetiche erano più propensi a dormire con la testa inserita tra le penne durante la notte. Inoltre, abbiamo trovato una correlazione positiva tra la quantità di cibo ingerito e le riserve energetiche alla cattura nei Beccafichi, ma non nelle Sterpazzole. Infine, non abbiamo trovato correlazioni significative dello stato ossidativo con il pattern del sonno e le riserve energetiche. Questi risultati suggeriscono che specie diverse potrebbero utilizzare strategie differenti per gestire la loro energia durante la sosta, e indicano che i passeriformi migratori posseggono adattamenti fisiologici per limitare i danni ossidativi prodotti durante la migrazione. Translated to Italian by Leonida Fusani and Andrea Ferretti

## Introduction

Twice a year, thousands of migratory bird species cover huge distances between their wintering and breeding grounds. Prior to migration, birds become hyperphagic and accumulate large energy reserves (King and Farner 1965; Odum 1960; McWilliams and Karasov 2005). During the crossings of large ecological barriers such as deserts or seas, birds perform multi-hour flights that can lead to depletion of their energy stores and to a generalized physiological stress, forcing them to make stopovers at the first suitable sites found after the barrier to rest and restore energy reserves (Schmaljohann et al. 2007). The physiological condition at arrival, in particular the extent of fat reserves, has a major influence on stopover behavior (Fusani et al. 2009; Goymann et al. 2010) and, time spent at the stopover site depends on the interplay between body condition at arrival (Dierschke and Delingat 2001; Goymann et al. 2010; Cohen et al. 2014; Smith and McWilliams 2014; Dossman et al. 2018) and the speed at which birds can restore their energy reserves (Lindström 2003; Gómez et al. 2017).

Migration is one of the most intense energy demanding life history stages, during which the highest mortality occurs (Sillett and Holmes 2002; Alerstam et al. 2003). Moreover, it is often associated with drastic physiological and behavioral changes other than the rapid gain and loss of energy stores. Several diurnal species, including a large proportion of passerine birds, become nocturnal migrants (Berthold 1973, 1996; Gwinner 1996). Flying at night and eating to accumulate energy reserves during the day limits the time available to sleep, which may become a constraint during this life history stage. Sleep is essential for all organisms (Shaw et al. 2002) and its deprivation may have dramatic consequences (Karni et al. 1994; Stickgold et al. 2000; Van Dongen et al. 2003), leading in the worst case to death (Rechtschaffen et al. 1983; Rechtschaffen and Bergmann 2002; Shaw et al. 2002). A large part of a bird’s life is spent sleeping (Toates 1980) but the function of this behavior is, in general, poorly understood. Several functions have been hypothesized, such as physiological restoration (Adam 1980; Reimund 1994; Mignot 2008), energy conservation (Berger 1975) and allocation (Schmidt 2014), clearance of metabolic waste products (Xie et al. 2013; Lim et al. 2013; Fultz et al. 2019), or memory consolidation (Maquet 2001; Stickgold et al. 2001). Among these, metabolic clearance has attracted considerable attention (Xie et al. 2013; Zhang et al. 2018). One group of molecules that might require clearance are the so-called reactive oxygen species (ROS) (Reimund 1994), atoms, or molecules with an unpaired electron. Given their chemical nature, these metabolites are highly reactive with biological molecules (i.e., proteins, lipids, and DNA) and can cause serious damage to the organism (Kregel and Zhang 2007; Cooper-Mullin and McWilliams 2016; Skrip and McWilliams 2016). Organisms can build antioxidant capacity (AOX), which can counteract ROS by reducing their reactivity, by upregulating antioxidant enzymes (enzymatic AOX) and by consuming dietary antioxidants (non-enzymatic AOX). According to the “free radical flux theory of sleep,” sleep clears ROS that have accumulated in the brain during wakefulness by reducing neurons’ activity and increasing enzymatic antioxidant mechanisms (Reimund 1994). Some evidence supporting the free radical flux theory has been found in *Drosophila*, where high ROS concentration in neurons directly triggers sleep (Hill et al. 2020). Moreover, the brain oxidative balance could be influenced by ROS produced in other tissues (e.g., liver, muscles, and red blood cells) and circulating antioxidants transported by the bloodstream. In this perspective, sleep may provide a direct antioxidant benefit to the brain and also play an important role in the maintenance of the oxidative balance in the periphery of the body. If sleep functions as, or allocates energy to, an antioxidant defense for the whole organism, it should be responsive to circulating ROS and thus may influence the oxidative status of the organism.

Although endurance migratory flights have been shown to increase ROS production (Costantini et al. 2008; Jenni-Eiermann et al. 2014), whether intense refueling bouts (Lindström 2003; Maggini et al. 2015) influence ROS concentration remains debated. Previous studies conducted on mammals showed that a high caloric intake is associated with high oxidative damage (Masoro 2000; Sohal and Weindruch 1996; Weindruch and Sohal 1997). Eikenaar et al. (2016) found that northern wheatears (*Oenanthe oenanthe*) that were experimentally fasted and refed and thus rapidly refueling did not increase oxidative damage, at least in part because of increased AOX. Skrip et al. (2015) also found that two species of free-living warblers that were fattening in preparation for fall migration increased AOX as they built fat stores; however, oxidative damage was also higher in fatter birds suggesting an inescapable hazard of using primarily fats as fuel. Moreover, sleep restriction experienced during migratory periods (Rattenborg et al. 2004) should reduce ROS clearance and lead to a further increase in circulating ROS levels. According to the hypothesis of an antioxidant function of sleep (Reimund 1994), sleeping during stopovers might help to reduce ROS concentration. A few field observations are in line with this hypothesis. Several European migratory species were reported to show diurnal sleep after crossing ecological barriers such as the Sahara Desert (Jenni-Eiermann et al. 2011) and the Mediterranean (Schwilch et al. 2002). For example, at Saharan stopover sites, migratory birds in good condition sleep during most of the day, despite having sufficient energy reserves to continue migration (Bairlein 1985; Biebach et al. 1986). The proportion of time spent sleeping/active, during both day and night, is strongly dependent on the physiological condition at arrival (Fusani et al. 2009; Ferretti et al. 2019b). Altogether, these studies suggest that migratory warblers, during both fall (Bairlein 1985; Biebach et al. 1986) and spring (Fusani et al. 2009; Ferretti et al. 2019b) migration, profit from stopover sites after crossing large ecological barriers to recover from sleep loss accumulated during non-stop flights. In addition, recent work from our group has shown that the posture adopted during sleep may influence energy conservation (Ferretti et al. 2019b). Birds can sleep in a tucked posture, in which the head is turned backward and tucked in the scapular feathers, or untucked, with the head pulled toward the body facing forward (Amlaner and Ball 1983). Lean migrating garden warblers (*Sylvia borin*) sleep mainly tucked in to reduce heat loss through the head, and this posture reduces conductance and, therefore, metabolic rate. By contrast, birds with large energy reserves expend more energy while sleeping untucked but react more quickly to threats. Thus, sleep posture preference during migration is the result of a trade-off between energy consumption and anti-predator vigilance (Ferretti et al. 2019b).

In the present study, we investigated the relationship between oxidative status, energy stores, food intake, and sleep in two migratory songbird species, the garden warbler and the whitethroat (*Sylvia communis*), at a Mediterranean stopover site during spring migration. Both species are long-distance migrants that cross similar large ecological barriers, and are abundant at our field site. Based on previous studies (Fusani et al. 2009; Goymann et al. 2010; Eikenaar and Schläfke 2013; Lupi et al. 2016), we expected birds with poor energy reserves to invest more time in energy recovery during the day and to sleep during most of the night with the head tucked. Birds with a large amount of energy reserves, on the contrary, should show a mainly untucked diurnal sleep pattern and higher nocturnal restlessness. Within this scenario, we hypothesized that there is a correlation between the oxidative status and the amount and type of sleep. Birds that land at the stopover site after an endurance flight are likely to have high ROS concentration. If sleep facilitates recovery from increased ROS, we predict that birds with higher levels of ROS will sleep longer, unless these birds also have a high antioxidant capacity. Moreover, birds with a high oxidative unbalance where pro-oxidant exceed antioxidants are expected to display a tucked sleep posture more often, which allows for deeper sleep and probably more efficient recovery from oxidative stress.

## Material and methods

### Study site and target species

This study was carried out on the island of Ponza in the Tyrrhenian Sea (40°55′ N, 12°58′ E). During spring migration, Ponza is an important stopover site for many European-African migratory birds that arrive after crossing the Mediterranean Sea, the second largest ecological barrier along their Spring migratory route. On Ponza, migrants that have just flown over sea can rest after their long nocturnal migratory flight.

Using mist nets, we caught 54 whitethroats and 63 garden warblers from March to May in 2015 and 2016. Both species are nocturnal trans-Saharan migrants with similar migratory routes, although garden warblers migrate slightly further north than whitethroats (Spina and Volponi 2008). The amount of subcutaneous fat (Kaiser 1993) and the size of the pectoral muscles were scored by an experienced ringer, who measured also the body mass following standardized European methods (Bairlein 1995). Within 3 min from capture, the brachial vein was punctured, and 100 µL of blood were collected using heparinized capillaries. The plasma was separated immediately after sampling by centrifugation and initially stored in liquid nitrogen and later at −80°C, until laboratory analysis.

### Sleep pattern and postural preference

After measurement and sampling, the birds were rapidly transported to the recording room and placed in custom-made fabric cages (50×25×30 cm) containing two perches at different heights. The cages were fitted inside custom-made ventilated soundproof boxes, to isolate the birds from external noise. The soundproof boxes were illuminated through a window and by a light system synchronized with the natural light/dark cycle. All birds were caught in the morning and placed in their cages by 12:00. They were kept there until the following sunrise. Birds were provided with 3 g of mealworms *Tenebrio molitor* and water *ad libitum*; the food bowl was removed at sunset and the remaining mealworms were weighed to measure food intake. During the housing period, behavior was recorded by infrared-sensitive cameras (700 line ccd camera; Handykam, Redruth, Cornwall, UK, 16 frm/s) connected to a recording system.

The video analysis was conducted using Solomon coder (version beta 16.06.26, developed by Péter 2016). Video-recordings were analyzed by focal, instantaneous sampling for 1 min each 5 min of recording. We divided the experimental period in two intervals: diurnal hours (from 1 p.m. until sunset) and nocturnal hours (from sunset until sunrise). We categorized behaviors into two main states: “Awake” and “Asleep.” A bird was coded as Asleep when it showed immobility for longer than 5 s and increased feather volume. The 5 s criterion is based on the fact that EEG signs of slow-wave sleep occur within a few seconds after onset of immobility in a sleep posture in a variety of avian species, including songbirds (see figures in Rattenborg et al. 2004; Lesku et al. 2012; Scriba et al. 2013; Tisdale et al. 2018). Birds were coded as Awake in all other cases. Asleep birds were further classified in two sub-states: in the “untucked” posture, the neck is retracted, and the head is pulled toward the body facing forward; in the “tucked” posture, the neck is turned backward, and the head tucked in the scapular feathers. The state “out of sight” was coded in the cases in which the bird was outside the surveilled area. To control for inter-observer variability, three entire days were analyzed independently by the three observers blind to the amount energy reserves data and inter-observer reliability was calculated by performing a Kruskal–Wallis test (*χ*^2^=0.136; *P* = 0.987).

### Measurement of plasma oxidative stress

To assess the oxidative balance, we used a protocol based on the simultaneous evaluation of the pro-oxidant status and antioxidant capacity. The pro-oxidant status was evaluated by means of a test that measures the free alcohoxil and hydroperoxyl radicals derived from hydroperoxides present in the sample (dROMs, Derivatives of Reactive Oxygen Metabolites, Diacron, Grosseto, Italy). After the reaction with a chromogen reagent, the metabolites produce a complex whose color intensity is directly proportional to their concentration. After incubation, the absorbance is read with a spectrophotometer at 500 nm and results are expressed in mmol/L of H_2_O_2_ equivalents. The anti-oxidant capacity (AOX) was measured using the OXY-Adsorbent test (Diacron) which quantifies the ability of the total serum or plasma anti-oxidant barrier (enzymatic and non-enzymatic) to cope with the oxidant action of hypochlorous acid (HOCl; oxidant of pathologic relevance in biological systems) by colorimetric determination. After the addition of the chromogen, the intensity of the colored complex, which is inversely related to the anti-oxidant power, is measured with a spectrophotometer at 500 nm. In this case, results are expressed in mmol/L of HClO neutralized. The methods are described in detail in Costantini and Dell’Omo (2006).

### Statistical analysis

Statistical analysis was conducted separately for each species; however, we analyzed the differences between species in energy reserves at arrival to verify that the two samples were homogeneous for this variable. As a proxy for energy reserves, we extracted the factor “condition” as the first component of a principal component analysis that included the variables fat score, muscle score, and body mass (Fusani et al. 2009; Ferretti et al. 2019b) (SPSS Statistics 25, IBM, NY, USA). We studied the relationship between energy reserves and oxidative stress markers using linear regression models (LMs). Moreover, we tested the relationship between proportion of food intake (grams intake/grams provided) and energy reserves using beta regression models. The use of the proportion instead of the absolute value was due to the bimodal distribution of food intake in garden warblers. Similarly, we tested the relationship between energy reserves, dROMs, and AOX on total sleep time and posture preference with beta regression models. We calculated condition both at capture and at release. Then, we used these values to calculate body condition change (condition at release − condition at capture). Moreover, we investigated the correlation between fat score, muscle score, and oxidative status—that is, pro-oxidant status, AOX, and the balance between them (dROMs/AOX * 1000)—using Spearman’s correlation tests. For the investigation of sleep patterns, the target variable was the ratio between total time spent asleep and the total time analyzed to control for differences in day/night length. With regard to sleep posture, the target variable was the ratio between the time spent in a given posture and the total time asleep to control for differences in total time spent sleeping. In order to use proportional data, the investigation of factors influencing sleep pattern and posture preference was conducted using beta regression models. Finally, we tested the relationship of total sleep time and the proportion of untucked sleep from caging to release with change in body condition using LMs.

## Results

### Physiological status at arrival

**Fig. 1:**
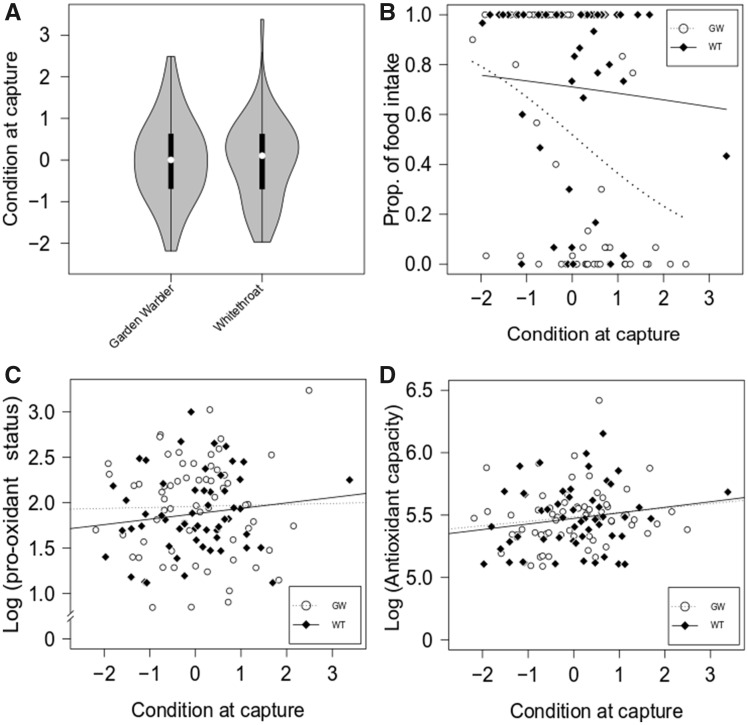
Distribution of condition at capture and its influence on proportion of food intake, pro-oxidant status, and antioxidant capacity in garden warbler and whitethroat. (**A**) The frequency distribution of condition at arrival was similar in garden warbler and whitethroat. Violin plots show the median (white dot), interquartile range (black bars), and distribution range (gray area) of the sample. The shape of the plot indicates the distribution of samples within the range. (**B**) Condition correlated with food intake (shown as proportion of available food) in garden warblers but not in whitethroats. The proportion was calculated as food intake (g) divided by food available (3 g). See also Supplementary Fig. S2. (**C**, **D**) There was no significant relationship between condition and pro-oxidant status (dROMs, expressed as mmol/L of H_2_O_2_ equivalents); C) and between condition and antioxidant capacity (AOX, expressed as mmol/L of HClO neutralized); D) at capture in any of our target species. In the plots, white dots and the dashed regression line represent garden warblers, whereas whitethroats are represented by black diamonds and the continuous regression line.

There was no difference in condition at arrival between species (LM: adjusted *R*^2^=−0.009, *P* = 1.000; Fig. 1). However, the two species differed in the distribution of food intake (LM: adjusted *R*^2^=0.089, *P* = 0.001; Supplementary Fig. S1). In whitethroats, the amount of food eaten was high regardless of condition (beta regression model: pseudo-*R*^2^=0.019, condition: *P* = 0.478; Fig. 1 and Supplementary Fig. S2). In garden warblers, only birds with low condition showed a high food intake, whereas individuals with high condition ate little (or none) of the food provided (beta regression model: pseudo-*R*^2^=0.279, condition: *P* < 0.001; Fig. 1 and Supplementary Fig. S2). In garden warblers and whitethroats we found no significant relationship between condition and either dROMs (LM: garden warbler, adjusted *R*^2^=−0.016, condition: *P* = 0.874; whitethroat, adjusted *R*^2^=−0.001, condition: *P* = 0.324) or AOX (LM: garden warbler, adjusted *R*^2^=0.008, condition: *P* = 0.229; whitethroat, adjusted *R*^2^ = 0.011, condition: *P* = 0.211) at capture (Fig. 1). Oxidative status also did not correlate with single components of the PCA (i.e., fat score and muscle score) when analyzed separately (Spearman’s correlation test; results are summarized in Supplementary Table S1 and Supplementary Figs. S3 and S4).

### Sleep behavior and condition

**Fig. 2:**
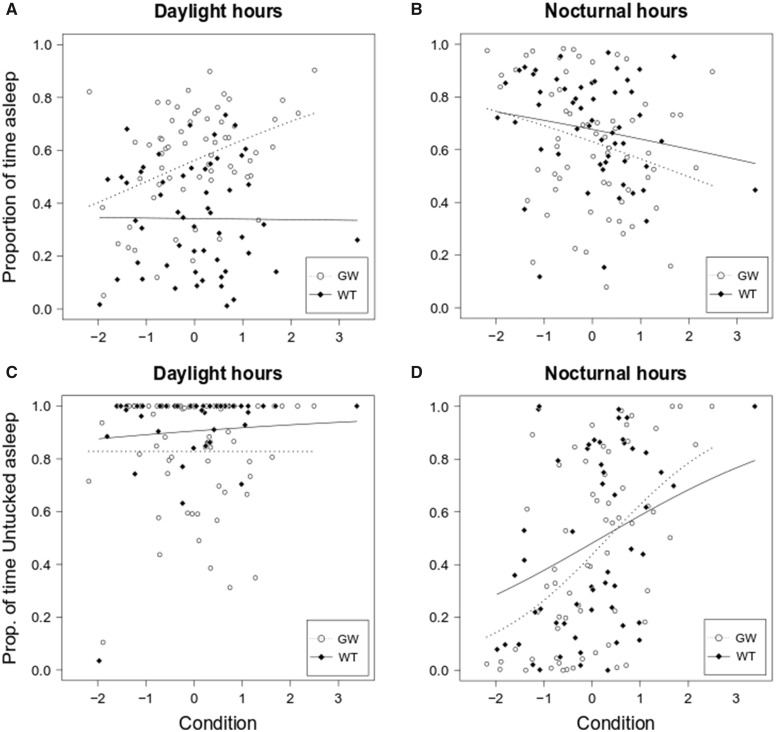
Relationship between condition and sleep in garden warblers and whitethroats. (**A**) In garden warblers, condition was positively associated with the amount of sleep, whereas in whitethroats this relationship was absent. (**B**) During the night, the amount of sleep was associated with condition in garden warblers but not in whitethroats, although the shape of the regression was similar. (**C**) Whitethroats and garden warblers showed a preference for the untucked sleep posture during the day but there was no association with condition. (**D**) During the night, sleep posture was strongly associated with condition in both species. In the plots, white dots and dashed regression lines represent garden warblers, black diamonds and continuous regression lines represent whitethroats.

During daytime, garden warblers showed a strong positive correlation between condition and the time spent sleeping (beta regression model: pseudo-*R*^2^=0.159, condition: *P* = 0.001). Such relationship was not found in whitethroats, which spent most of daytime awake, regardless on their energy reserves (beta regression model: pseudo-*R*^2^<0.001, condition: *P* = 0.942) (Fig. 2). At night, the amount of time spent sleeping was inversely related to condition in garden warbler (beta regression model: pseudo-*R*^2^=0.082, condition: *P* = 0.028) but not in whitethroats (beta regression model: pseudo-*R*^2^=0.038, condition: *P* = 0.163), although the slopes of the regression lines look similar (Fig. 2).

Despite some differences in the sleep patterns between our study species, they showed similar results in relation to sleep posture preference. Regardless of condition, both species showed a clear preference for the untucked sleep posture during daylight hours (beta regression model; garden warbler, pseudo-*R*^2^=0.001, condition: *P* = 0.996; whitethroat, pseudo-*R*^2^=0.055, condition: *P* = 0.260) (Fig. 2). During the night, sleep posture was dependent on condition in both species: the untucked posture was adopted more frequently when condition was high (beta regression model; garden warbler, pseudo-*R*^2^=0.372, condition: *P* < 0.001; whitethroat, pseudo-*R*^2^=0.146, condition: *P* = 0.008; Fig. 2).

### Oxidative status: dROMs and AOX

**Fig. 3:**
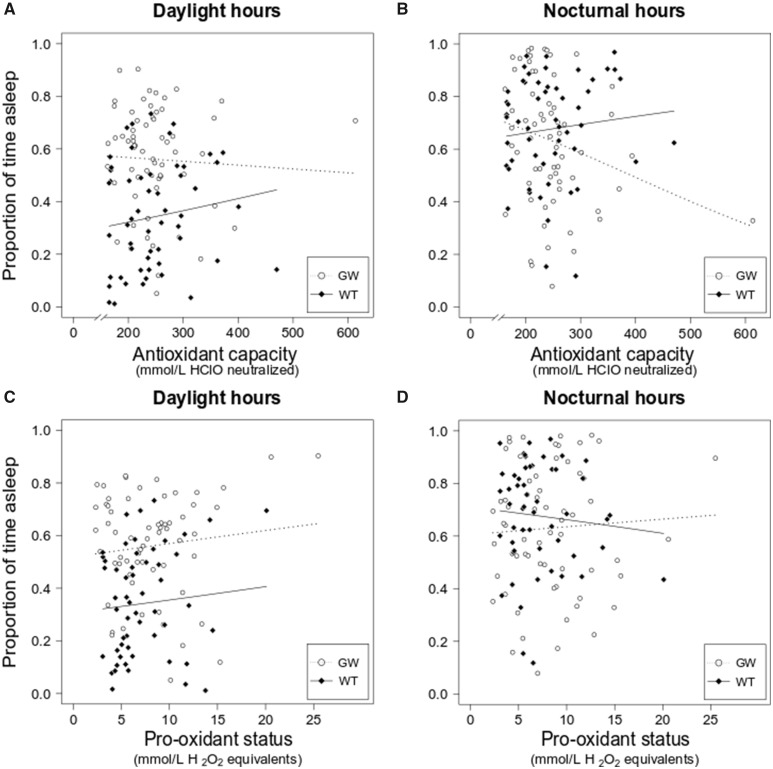
Relationship between antioxidant capacity and pro-oxidant status and sleep patterns in garden warblers and whitethroats. (**A**) Our target species showed different diurnal sleep patterns, which were not influenced by AOX levels. (**B**) During the night, the amount of sleep was affected by AOX in garden warblers but not in whitethroats. (**C**) Pro-oxidant status did not affect sleep during the day. (**D**) Nocturnal sleep patterns were not affected by pro-oxidant status in both species. In the plots, white dots and dashed regression lines represent garden warblers, black diamonds, and continuous regression lines represent whitethroats.

We did not find global differences between species in markers of oxidative status at capture (LM: AOX, adjusted *R*^2^: −0.008, *P* = 0.792; dROMs, adjusted *R*^2^: −0.002, *P* = 0.381). During the day, there were no effects of AOX or dROMs levels on sleep in both species (Table 1 and Fig. 3). During the night, we found a significant effect of AOX on nocturnal sleep in garden warblers but not in whitethroats (Table 1 and Fig. 3). The pro-oxidant status as indicated by dROMs did not affect nocturnal sleep in either species (Table 1 and Fig. 3). Finally, AOX and dROMs had no effects on posture preference (Table 1 and Supplementary Fig. S5). There were some extreme values of AOX and dROMs. As these values fall within the physiological range, we had no reason to exclude them from our analysis. However, we estimated the influence of each potential outlier—meant as the difference in intercept and estimate between the full model and the model excluding the extreme value—on each model using the “dfbetas” function in R. We reported the results in Supplementary Table S2. In garden warblers, the effect of AOX on the amount of sleep during the night was dependent on the most extreme AOX value, as the significance of the test disappeared after removing this data point.

**Table 1:** Outcome of beta regression models on the whole dataset testing for differences in sleep behavior depending on dROMs (marker of pro-oxidant status) and AOX (antioxidant capacity)

		Daylight hours				Nocturnal hours			
		Total sleep		Untucked		Total sleep		Untucked	
		Pseudo-*R*^2^	*P*-value	Pseudo-*R*^2^	*P*-value	Pseudo-*R*^2^	*P*-value	Pseudo-*R*^2^	*P*-value
Garden warbler	AOX	0.003	0.687	0.001	0.916	**0.062**	**0.041**	0.016	0.327
	dROMs	0.016	0.365	0.001	0.893	0.003	0.655	0.002	0.725
Whitethroat	AOX	0.023	0.271	0.001	0.906	0.015	0.395	0.008	0.539
	dROMs	0.007	0.533	0.002	0.851	0.008	0.506	0.001	0.873

Total sleep refers to the proportion of total time spent asleep, whereas untucked refers to the proportion of sleep time spent in the untucked posture. Statistically significant effects are outlined in bold typeface.

### Body condition change

**Fig. 4:**
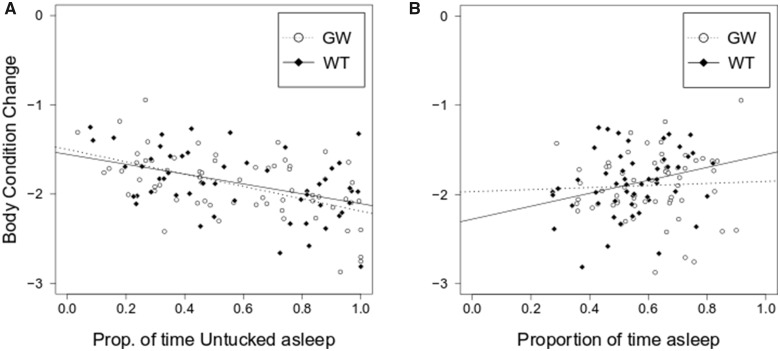
Relationship between sleep parameters and body condition change in garden warblers and whitethroats. (**A**) Body condition change was negatively correlated with sleep posture preference in both species. (**B**) The total amount of sleep positively correlated with body condition change in whitethroats, but not in garden warblers.

In both species, the largest change in body condition occurred in birds with the highest proportion of total untucked sleep (LM: garden warbler, adjusted *R*^2^=0.272, untucked: *P* < 0.001; whitethroat, adjusted *R*^2^=0.177, untucked: *P* = 0.001, Fig. 4). Moreover, body condition change was positively correlated with the total amount of time spent asleep in whitethroats (LM: adjusted *R*^2^=0.057, sleep time: *P* = 0.045, Fig. 4), but was not in garden warblers (LM; adjusted *R*^2^=−0.014, sleep time: *P* = 0.720, Fig. 4).

## Discussion

In this study, we found that garden warblers and whitethroats showed the same posture preference patterns in relation to their energy reserves, indicating that the use of the tucked sleep posture in energetically challenged individuals is a common energy saving strategy. This confirms a thermoregulatory function of sleep posture preference in a second migratory species as already suggested for non-migratory species (Midtgård 1978; Reebs 1986; Pavlovic et al. 2019). However, further studies are needed to clarify if energy saved through reducing conductance (Ferretti et al. 2019b) is exclusively invested in condition maintenance or partially reallocated toward sleep-coupled processes that benefit the organism (Schmidt 2014).

Despite having very similar amounts of energy reserves and oxidative status at arrival, garden warblers and whitethroats showed some differences in the way energy reserves affect their sleep behavior during spring stopover. As already reported in Ferretti et al. (2019b), the amount of energy reserves was the main factor affecting the amount of time spent asleep in garden warblers. In contrast, in whitethroats the amount of sleep was not correlated with the amount of energy reserves, neither during the day nor during the night. Moreover, the total amount of sleep correlated with the change in amount of energy reserves in whitethroats but not in garden warblers. These two species are known to manage their activity at stopover sites differently: at a desert stopover site, whitethroats were most commonly observed foraging, while garden warblers were found sleeping on several occasions (Jenni-Eiermann et al. 2011). These converging findings between caged and free-living birds provide further evidence about the reliability of the results obtained with temporarily caged birds to reveal physiological and behavioral adaptations of migratory birds (Fusani et al. 2009; Goymann et al. 2010; Eikenaar et al. 2014).

Besides the differences in sleep pattern, we also found differences in feeding behavior between the two species. Whitethroats showed a high food intake regardless of their energy reserves, which may indicate that this species needs to maximize energy intake through an intense exploitation of the stopover site before resuming migration. On the contrary, in garden warblers the amount of food intake was strongly dependent on amount of energy reserves, confirming the results of previous studies (Goymann et al. 2017; Lupi et al. 2017). These findings further indicate different stopover strategies in our target species. In the present study, food available to birds corresponded to the average amount eaten by birds in poor condition caught in Ponza in spring (Ferretti et al. 2019a). We cannot rule out, however, that results might change with different diets or food regimes.

During migration, birds might need to cope with an increased production of ROS due to high metabolic rate that occurs during flapping flight (Costantini et al. 2008). Although some studies have shown a relationship between oxidative balance and energy reserves during autumn migration (Jenni-Eiermann et al. 2014; Skrip et al. 2015; Eikenaar et al. 2020), we did not find any association between measures of oxidative status and condition at capture, no matter whether we considered the extracted variable condition or each component of condition (fat score or muscle score) separately, confirming the results of a previous study on spring-migrating garden warblers caught on Ponza (Skrip et al. 2015). According to the “free radical flux theory,” sleep functions as an antioxidant barrier that clears ROS accumulation from the brain (Reimund 1994). In our study, pro-oxidant status as measured in blood did not correlate with either sleep pattern or sleep posture preference in both species. These findings suggest that circulating pro-oxidants do not trigger sleep, as expected according to its antioxidant function. However, plasma pro-oxidants might be a good marker for the general ROS circulating level due to cellular metabolism in different tissues (i.e., muscles, liver, and red blood cells), but whether they are also a good measure of brain oxidative status, which is thought to induce sleep (Reimund 1994), remains to be demonstrated. An alternative explanation might be that stress resulting from capture and handling have overridden the relationships between the oxidative status measured soon after capture and behavior measured in the cages. However, our series of studies on a number of species on Ponza, together with studies of other groups at other stopover sites (Eikenaar et al. 2019), show a robust association between the results found in captivity and those reported from free-living birds (Jenni-Eiermann et al. 2011). Although further studies are needed to understand how oxidative status relates to sleep patterns, the lack of a significant relationship between these factors found in the current study might be related to unknown aspects, for example, AOX levels prior to departure. Indeed, the accumulation of enough antioxidant molecules prior to departure could counteract ROS production during the following endurance flight (Costantini et al. 2007), keeping oxidative damage below levels that would trigger the antioxidant function of sleep. This hypothesis is supported by the preference for fruits with high dietary antioxidant content shown by migrants during stopover (Alan and McWilliams 2013; Bolser et al. 2013; Schaefer et al. 2014; Cooper-Mullin and McWilliams 2016).

The amount of energy reserves (Fusani et al. 2009; Goymann et al. 2010) and fuel deposition rate (Lindström 2003; Schaub et al. 2008) are fundamental drivers of stopover decisions (Schmaljohann and Eikenaar 2017). Departure from stopover is determined by several factors such as condition at arrival (Dierschke and Delingat 2001; Goymann et al. 2010), hormonal levels (Goymann et al. 2017; Eikenaar et al. 2017), food availability (Fusani et al. 2011; Lupi et al. 2017), and predation risk (Ydenberg et al. 2002; Dierschke 2003). Species that fly along the same migratory path may follow different refueling strategies during their journey (Hedenström and Alerstam 1997), as indicated by their residual flight range—the estimated residual distance that the bird can cover according to its energy reserves (Pilastro and Spina 1997). Regardless of their condition, whitethroats showed a high intensity of refueling coupled with a low proportion of time spent sleeping during the day. In contrast, garden warblers seem to have a more condition-dependent refueling strategy which leads them to refuel only when strictly necessary. These differences might depend on a number of factors, such as differences in total migratory distance or simply different migratory strategies (Hedenström and Alerstam 1997). Differences in sleep patterns were found also during the night: in whitethroats, the amount of nocturnal sleep was not influenced by the amount of energy reserves as found in garden warblers, which was expected based on previous studies on migratory disposition in captive migrants (Fusani et al. 2009; Lupi et al. 2016; Schmaljohann and Eikenaar 2017).

In summary, our results confirm the key role of energy reserves in determining behavior during stopover. Although further investigations are required to better understand the use of sleep in energy management during stopover and whether sleep is affected exclusively by the extent of energy reserves or by the interaction between energy reserves and food intake, our findings encourage novel perspectives on avian migration. Moreover, the lack of influence of pro-oxidant status on stopover behavior suggests the presence of physiological adaptations that reduce the expected overproduction of ROS during migration. This is particularly important for birds seeking to experience a rapid accumulation of energy reserves. Indeed, there is some evidence that ROS induce resistance to insulin—which converts food into energy reserves (Hoehn et al. 2009; Mouzannar et al. 2011) and thus slow down the re-fueling process in migrating birds (Totzke et al. 1997, 1998; Totzke and Bairlein 1998). Therefore, the investigation of physiological mechanisms involved in the mitigation of oxidative damage during migration will be important for understanding adaptations to this life-history stage. In addition to the direct benefit for avian research, the investigation of such mechanisms may be exported to mammalian models and used to improve our understanding of metabolic syndromes in human (Bairlein 2002; Goymann et al. 2017).

## Ethical permits

All experimental procedures including the permission to trap and temporarily hold birds in temporary captivity were authorized by the Regional Government (Determina Regione Lazio N. G02278 of 6 June 2015) in accordance with EU and Italian laws, and were communicated to, and performed according to the guidelines of, the Ethic and Animal Protection Committee (ETK) of the University of Veterinary Medicine, Vienna.

## Data availability

Data deposited in the Phaidra Digital Repository: https://phaidra.univie.ac.at/o:1056786.

## Author contributions

A.F., S.R.M., and L.F. conceived the study. A.F. and M.C. conducted the experimental work. A.F. and I.M. analyzed the data. A.F., L.F., N.C.R., S.R.M., and I.M. wrote the manuscript.

## Supplementary Material

obaa010_Supplementary_Data

## References

[obaa010-B1] Adam K. 1980 Sleep as a restorative process and a theory to explain why In: McConnell PS, Boer GJ, Romijn HJ, Van De Poll NE,, Corner MA, editors. Progress in brain research. Amsterdam: Elsevier p. 289–305. 10.1016/S0079-6123(08)60070-9 7005947

[obaa010-B2] Alan RR, McWilliams SR. 2013 Oxidative stress, circulating antioxidants, and dietary preferences in songbirds. Comp Biochem Physiol B Biochem Mol Biol 164:185–93. 23270695 10.1016/j.cbpb.2012.12.005

[obaa010-B3] Alerstam T, Hedenström A, Åkesson S. 2003 Long-distance migration: evolution and determinants. Oikos 103:247–60.

[obaa010-B4] Amlaner CJ Jr, Ball NJ. 1983 A synthesis of sleep in wild birds. Behaviour 87:85–119.

[obaa010-B5] Bairlein F. 1985 Body weights and fat deposition of Palaearctic passerine migrants in the central Sahara. Oecologia 66:141–6. 28310826 10.1007/BF00378566

[obaa010-B6] Bairlein F. 1995 Manual of field methods. Wilhelmshaven, Germany: European-African songbird migration network.

[obaa010-B7] Bairlein F. 2002 How to get fat: nutritional mechanisms of seasonal fat accumulation in migratory songbirds. Naturwissenschaften 89:1–10. 12008967 10.1007/s00114-001-0279-6

[obaa010-B8] Berger RJ. 1975 Bioenergetic functions of sleep and activity rhythms and their possible relevance to aging In: Thorbecke GJ, editor. Biology of aging and development. Boston (MA): Springer US p. 191–202. 162800

[obaa010-B9] Berthold P. 1973 Relationships between migratory restlessness and migration distance in six *Sylvia* species. Ibis 115:594–9.

[obaa010-B10] Berthold P. 1996 Control of bird migration. London: Chapman & Hall.

[obaa010-B11] Biebach H, Friedrich W, Heine G. 1986 Interaction of bodymass, fat, foraging and stopover period in trans-Sahara migrating passerine birds. Oecologia 69:370–9. 28311339 10.1007/BF00377059

[obaa010-B12] Bolser JA, Alan RR, Smith AD, Li L, Seeram NP, McWilliams SR. 2013 Birds select fruits with more anthocyanins and phenolic compounds during autumn migration. Wilson J Ornithol 125:97–108.

[obaa010-B13] Cohen EB, Moore FR, Fischer RA. 2014 Fuel stores, time of spring, and movement behavior influence stopover duration of Red-eyed Vireo *Vireo olivaceus*. J Ornithol 155:785–92.

[obaa010-B14] Cooper-Mullin C, McWilliams SR. 2016 The role of the antioxidant system during intense endurance exercise: lessons from migrating birds. J Exp Biol 219:3684–95. 27903627 10.1242/jeb.123992

[obaa010-B15] Costantini D, Cardinale M, Carere C. 2007 Oxidative damage and anti-oxidant capacity in two migratory bird species at a stop-over site. Comp Biochem Physiol C 144:363–71. 10.1016/j.cbpc.2006.11.005 17218158

[obaa010-B16] Costantini D, Dell’Ariccia G, Lipp H-P. 2008 Long flights and age affect oxidative status of homing pigeons (*Columba livia*). J Exp Biol 211:377–81. 18203993 10.1242/jeb.012856

[obaa010-B17] Costantini D, Dell’Omo G. 2006 Environmental and genetic components of oxidative stress in wild kestrel nestlings (*Falco tinnunculus*). J Comp Physiol B 176:575–9. 16598486 10.1007/s00360-006-0080-0

[obaa010-B18] Dierschke V. 2003 Predation hazard during migratory stopover: are light or heavy birds under risk?. J Avian Biol 34:24–9.

[obaa010-B19] Dierschke V, Delingat J. 2001 Stopover behaviour and departure decision of northern wheatears, *Oenanthe oenanthe*, facing different onward non-stop flight distances. Behav Ecol Sociobiol 50:535–45.

[obaa010-B20] Dossman BC, Matthews SN, Rodewald PG. 2018 An experimental examination of the influence of energetic condition on the stopover behavior of a Nearctic–Neotropical migratory songbird, the American Redstart (*Setophaga ruticilla*). Auk 135:91–100, 10.

[obaa010-B21] Eikenaar C, Hegemann A, Packmor F, Kleugden I, Isaksson C. 2020 Not just fuel: energy stores are correlated with immune function and oxidative damage in a long-distance migrant. Curr Zool 66:21–8. 32467701 10.1093/cz/zoz009 PMC7245008

[obaa010-B22] Eikenaar C, Hessler S, Fischer S, Bairlein F. 2019 An exception to the rule: captivity does not stress wild migrating northern wheatears. Gen Comp Endocrinol 275:25–9. 30753841 10.1016/j.ygcen.2019.02.010

[obaa010-B23] Eikenaar C, Jönsson J, Fritzsch A, Wang H-L, Isaksson C. 2016 Migratory refueling affects non-enzymatic antioxidant capacity, but does not increase lipid peroxidation. Physiol Behav 158:26–32. 26921098 10.1016/j.physbeh.2016.02.033

[obaa010-B24] Eikenaar C, Klinner T, Szostek KL, Bairlein F. 2014 Migratory restlessness in captive individuals predicts actual departure in the wild. Biol Lett 10:20140154. 24718095 10.1098/rsbl.2014.0154 PMC4013702

[obaa010-B25] Eikenaar C, Müller F, Leutgeb C, Hessler S, Lebus K, Taylor PD, Schmaljohann H. 2017 Corticosterone and timing of migratory departure in a songbird. Proc R Soc Lond B 284:20162300. 10.1098/rspb.2016.2300 PMC5247501 28077768

[obaa010-B26] Eikenaar C, Schläfke JL. 2013 Size and accumulation of fuel reserves at stopover predict nocturnal restlessness in a migratory bird. Biol Lett 9:20130712. 24132097 10.1098/rsbl.2013.0712 PMC3871357

[obaa010-B27] Ferretti A, Maggini I, Lupi S, Cardinale M, Fusani L. 2019a. The amount of available food affects diurnal locomotor activity in migratory songbirds during stopover. Sci Rep 9:19027. 31836848 10.1038/s41598-019-55404-3 PMC6910983

[obaa010-B28] Ferretti A, Rattenborg NC, Ruf T, McWilliams SR, Cardinale M, Fusani L. 2019b. Sleeping unsafely tucked in to conserve energy in a nocturnal migratory songbird. Curr Biol 29:2766–72.e4. 31430467 10.1016/j.cub.2019.07.028

[obaa010-B29] Fultz NE, Bonmassar G, Setsompop K, Stickgold RA, Rosen BR, Polimeni JR, Lewis LD. 2019 Coupled electrophysiological, hemodynamic, and cerebrospinal fluid oscillations in human sleep. Science 366:628–31. 31672896 10.1126/science.aax5440 PMC7309589

[obaa010-B30] Fusani L, Cardinale M, Carere C, Goymann W. 2009 Stopover decision during migration: physiological conditions predict nocturnal restlessness in wild passerines. Biol Lett 5:302–5. 19324648 10.1098/rsbl.2008.0755 PMC2679912

[obaa010-B31] Fusani L, Cardinale M, Schwabl I, Goymann W. 2011 Food availability but not melatonin affects nocturnal restlessness in a wild migrating passerine. Horm Behav 59:187–92. 21110977 10.1016/j.yhbeh.2010.11.013

[obaa010-B32] Gómez C, Bayly NJ, Norris DR, Mackenzie SA, Rosenberg KV, Taylor PD, Hobson KA, Daniel Cadena C. 2017 Fuel loads acquired at a stopover site influence the pace of intercontinental migration in a boreal songbird. Sci Rep 7:3405. 28611372 10.1038/s41598-017-03503-4 PMC5469819

[obaa010-B33] Goymann W, Lupi S, Kaiya H, Cardinale M, Fusani L. 2017 Ghrelin affects stopover decisions and food intake in a long-distance migrant. Proc Natl Acad Sci U S A 114:1946–51. 28167792 10.1073/pnas.1619565114 PMC5338360

[obaa010-B34] Goymann W, Spina F, Ferri A, Fusani L. 2010 Body fat influences departure from stopover sites in migratory birds: evidence from whole-island telemetry. Biol Lett 6:478–81. 20164077 10.1098/rsbl.2009.1028 PMC2936206

[obaa010-B35] Gwinner E. 1996 Circadian and circannual programmes in avian migration. J Exp Biol 199:39–48. 9317295 10.1242/jeb.199.1.39

[obaa010-B36] Hedenström A, Alerstam T. 1997 Optimum fuel loads in migratory birds: distinguishing between time and energy minimization. J Theor Biol 189:227–34. 9441816 10.1006/jtbi.1997.0505

[obaa010-B37] Hill VM, O’Connor RM, Shirasu-Hiza M. 2020 Tired and stressed: examining the need for sleep. Eur J Neurosci 51:494–15. 30295966 10.1111/ejn.14197 PMC6453762

[obaa010-B38] Hoehn KL, Salmon AB, Hohnen-Behrens C, Turner N, Hoy AJ, Maghzal GJ, Stocker R, Van Remmen H, Kraegen EW, Cooney GJ, et al 2009 Insulin resistance is a cellular antioxidant defense mechanism. Proc Natl Acad Sci U S A 106:17787–92. 19805130 10.1073/pnas.0902380106 PMC2764908

[obaa010-B39] Jenni-Eiermann S, Almasi B, Maggini I, Salewski V, Bruderer B, Liechti F, Jenni L. 2011 Numbers, foraging and refuelling of passerine migrants at a stopover site in the Western Sahara: diverse strategies to cross a desert. J Ornithol 152:113–28.

[obaa010-B40] Jenni-Eiermann S, Jenni L, Smith S, Costantini D. 2014 Oxidative stress in endurance flight: an unconsidered factor in bird migration. PLoS ONE 9:e97650. 24830743 10.1371/journal.pone.0097650 PMC4022615

[obaa010-B41] Kaiser A. 1993 A new multi-category classification of subcutaneous fat deposits of songbirds. J Field Ornithol 64:246–55.

[obaa010-B42] Karni A, Tanne D, Rubenstein BS, Askenasy JJM, Sagi D. 1994 Dependence on REM sleep of overnight improvement of a perceptual skill. Science 265:679–82. 8036518 10.1126/science.8036518

[obaa010-B43] King JR, Farner DS. 1965 Studies of fat deposition in migratory birds. Ann N Y Acad Sci 131:422–40. 5216979 10.1111/j.1749-6632.1965.tb34808.x

[obaa010-B44] Kregel KC, Zhang HJ. 2007 An integrated view of oxidative stress in aging: basic mechanisms, functional effects, and pathological considerations. Am J Physiol Reg Int Comp Physiol 292:R18–36. 10.1152/ajpregu.00327.2006 16917020

[obaa010-B45] Lesku JA, Rattenborg NC, Valcu M, Vyssotski AL, Kuhn S, Kuemmeth F, Heidrich W, Kempenaers B. 2012 Adaptive sleep loss in polygynous pectoral sandpipers. Science 337:1654–8. 22878501 10.1126/science.1220939

[obaa010-B46] Lim ASP, Kowgier M, Yu L, Buchman AS, Bennett DA. 2013 Sleep fragmentation and the risk of incident Alzheimer’s disease and cognitive decline in older persons. Sleep 36:1027–32. 23814339 10.5665/sleep.2802 PMC3669060

[obaa010-B47] Lindström Å. 2003 Fuel deposition rates in migrating birds: causes, constraints and consequences In: Berthold P, Gwinner E, Sonnenschein E, editors. Avian migration. Berlin, Heidelberg: Springer p. 307–20.

[obaa010-B48] Lupi S, Goymann W, Cardinale M, Fusani L. 2016 Physiological conditions influence stopover behaviour of short-distance migratory passerines. J Ornithol 157:583–9.

[obaa010-B49] Lupi S, Maggini I, Goymann W, Cardinale M, Rojas Mora A, Fusani L. 2017 Effects of body condition and food intake on stop-over decisions in garden warblers and European Robins during spring migration. J Ornithol 158:989–99.

[obaa010-B50] Maggini I, Hama F, Robson D, Rguibi Idrissi H, Bairlein F, Gargallo G. 2015 Foraging behavior of three species of songbirds during stopover in southeastern Morocco during spring migration. J Field Ornithol 86:266–76.

[obaa010-B51] Maquet P. 2001 The role of sleep in learning and memory. Science 294:1048–52. 11691982 10.1126/science.1062856

[obaa010-B52] Masoro EJ. 2000 Caloric restriction and aging: an update. Exp Gerontol 35:299–305. 10832051 10.1016/s0531-5565(00)00084-x

[obaa010-B53] McWilliams SR, Karasov WH. 2005 Migration takes guts. Digestive physiology of migratory birds and its ecological significance In: Marra P, Greenberg R, editors. Birds of two worlds. Washington (DC): Smithsonian Inst Press p. 67–79.

[obaa010-B54] Midtgård U. 1978 Resting postures of the Mallard *Anas platyrhynchos*. Ornis Scand 9:214–9.

[obaa010-B55] Mignot E. 2008 Why we sleep: the temporal organization of recovery. PLoS Biol 6:e106. 18447584 10.1371/journal.pbio.0060106 PMC2689703

[obaa010-B56] Mouzannar R, McCafferty J, Benedetto G, Richardson C. 2011 Transcriptional and phospho-proteomic screens reveal stem cell activation of insulin-resistance and transformation pathways following a single minimally toxic episode of ROS. Int J Genomics Proteomics 2:34–49. 21743783 PMC3131088

[obaa010-B57] Odum EP. 1960 Premigratory hyperphagia in birds. Am J Clin Nutr 8:621–9.

[obaa010-B58] Pavlovic G, Weston MA, Symonds MRE. 2019 Morphology and geography predict the use of heat conservation behaviours across birds. Funct Ecol 33:286–96.

[obaa010-B59] Péter A. 2016 Solomon Coder: a simple solution for behavior coding. Ed. 16.06.26 (https://solomoncoder.com/).

[obaa010-B60] Pilastro A, Spina F. 1997 Ecological and morphological correlates of residual fat reserves in passerine migrants at their spring arrival in southern Europe. J Avian Biol 28:309–18.

[obaa010-B61] Rattenborg NC, Mandt BH, Obermeyer WH, Winsauer PJ, Huber R, Wikelski M, Benca RM. 2004 Migratory sleeplessness in the white-crowned sparrow (*Zonotrichia leucophrys gambelii*). PLoS Biol 2:e212. 15252455 10.1371/journal.pbio.0020212 PMC449897

[obaa010-B62] Rechtschaffen A, Bergmann BM. 2002 Sleep deprivation in the rat: an update of the 1989. Paper Sleep 25:18–24. 11833856 10.1093/sleep/25.1.18

[obaa010-B63] Rechtschaffen A, Gilliland MA, Bergmann BM, Winter JB. 1983 Physiological correlates of prolonged sleep deprivation in rats. Science 221:182–4. 6857280 10.1126/science.6857280

[obaa010-B64] Reebs SG. 1986 Sleeping behavior of black-billed magpies under a wide range of temperatures. Condor 88:524–6.

[obaa010-B65] Reimund E. 1994 The free radical flux theory of sleep. Med Hypotheses 43:231–3. 7838006 10.1016/0306-9877(94)90071-x

[obaa010-B66] Schaefer HM, Valido A, Jordano P. 2014 Birds see the true colours of fruits to live off the fat of the land. Proc R Soc Lond B 281:20132516. 10.1098/rspb.2013.2516 PMC3896014 24403330

[obaa010-B67] Schaub M, Jenni L, Bairlein F. 2008 Fuel stores, fuel accumulation, and the decision to depart from a migration stopover site. Behav Ecol 19:657–66.

[obaa010-B68] Schmaljohann H, Eikenaar C. 2017 How do energy stores and changes in these affect departure decisions by migratory birds? A critical view on stopover ecology studies and some future perspectives. J Comp Physiol A 203:411–29. 10.1007/s00359-017-1166-8 28332031

[obaa010-B69] Schmaljohann H, Liechti F, Bruderer B. 2007 An addendum to ‘songbird migration across the Sahara: the non-stop hypothesis rejected!’ Proc R Soc Lond B 274:1919–20. 10.1098/rspb.2007.0188 PMC2270925 17519192

[obaa010-B70] Schmidt MH. 2014 The energy allocation function of sleep: a unifying theory of sleep, torpor, and continuous wakefulness. Neurosci Biobehav Rev 47:122–53. 25117535 10.1016/j.neubiorev.2014.08.001

[obaa010-B71] Schwilch R, Piersma T, Holmgren NMA, Jenni L. 2002 Do migratory birds need a nap after a long non-stop flight? Ardea 90:149–54.

[obaa010-B72] Scriba MF, Ducrest A-L, Henry I, Vyssotski AL, Rattenborg NC, Roulin A. 2013 Linking melanism to brain development: expression of a melanism-related gene in barn owl feather follicles covaries with sleep ontogeny. Front Zool 10:42. 23886007 10.1186/1742-9994-10-42 PMC3734112

[obaa010-B73] Shaw PJ, Tononi G, Greenspan RJ, Robinson DF. 2002 Stress response genes protect against lethal effects of sleep deprivation in *Drosophila*. Nature 417:287–91. 12015603 10.1038/417287a

[obaa010-B74] Sillett TS, Holmes RT. 2002 Variation in survivorship of a migratory songbird throughout its annual cycle. J Anim Ecol 71:296–308.

[obaa010-B75] Skrip MM, Bauchinger U, Goymann W, Fusani L, Cardinale M, Alan RR, McWilliams SR. 2015 Migrating songbirds on stopover prepare for, and recover from, oxidative challenges posed by long-distance flight. Ecol Evol 5:3198–209. 26355277 10.1002/ece3.1601 PMC4559061

[obaa010-B76] Skrip MM, McWilliams SR. 2016 Oxidative balance in birds: an atoms-to-organisms-to-ecology primer for ornithologists. J Field Ornithol 87:1–20.

[obaa010-B77] Smith AD, McWilliams SR. 2014 What to do when stopping over: behavioral decisions of a migrating songbird during stopover are dictated by initial change in their body condition and mediated by key environmental conditions. Behav Ecol 25:1423–35.

[obaa010-B78] Sohal RS, Weindruch R. 1996 Oxidative stress, caloric restriction, and ageing. Science 273:59–63. 8658196 10.1126/science.273.5271.59 PMC2987625

[obaa010-B79] Spina F, Volponi S. 2008. Atlante della Migrazioni degli Uccelli in Italia. II. Passeriformi., Ozzano dell’Emilia (Bologna), Ministero dell’Ambiente e della Tutela del Territorio e del Mare, Istituto Superiore per la Protezione e la Ricerca Ambientale (ISPRA).

[obaa010-B80] Stickgold R, Hobson JA, Fosse R, Fosse M. 2001 Sleep, learning, and dreams: off-line memory reprocessing. Science 294:1052–7. 11691983 10.1126/science.1063530

[obaa010-B81] Stickgold R, James L, Hobson JA. 2000 Visual discrimination learning requires sleep after training. Nat Neurosci 3:1237–8. 11100141 10.1038/81756

[obaa010-B82] Tisdale RK, Lesku JA, Beckers GJL, Vyssotski AL, Rattenborg NC. 2018 The low-down on sleeping down low: pigeons shift to lighter forms of sleep when sleeping near the ground. J Exp Biol 221:jeb182634. 30287589 10.1242/jeb.182634

[obaa010-B83] Toates FM. 1980 Animal behaviour: a systems approach. New York: Wiley.

[obaa010-B84] Totzke U, Bairlein F. 1998 The body mass cycle of the migratory garden warbler (*Sylvia borin*) is associated with changes of basal plasma metabolite levels. Comp Biochem Physiol A 121:127–33.

[obaa010-B85] Totzke U, Hübinger A, Bairlein F. 1997 A role for pancreatic hormones in the regulation of autumnal fat deposition of the garden warbler (*Sylvia borin*)? Gen Comp Endocrinol 107:166–71. 9245524 10.1006/gcen.1997.6909

[obaa010-B86] Totzke U, Hübinger A, Bairlein F. 1998 Glucose utilization rate and pancreatic hormone response to oral glucose loads are influenced by the migratory condition and fasting in the garden warbler (*Sylvia borin*). J Endocrinol 158:191–6. 9771462 10.1677/joe.0.1580191

[obaa010-B87] Van Dongen HP, Maislin G, Mullington JM, Dinges DF. 2003 The cumulative cost of additional wakefulness: dose–response effects on neurobehavioral functions and sleep physiology from chronic sleep restriction and total sleep deprivation. Sleep 26:117–26. 12683469 10.1093/sleep/26.2.117

[obaa010-B88] Weindruch R, Sohal RS. 1997 Seminars in medicine of the Beth Israel Deaconess Medical Center. Caloric intake and aging. N Engl J Med 337:986–94. 9309105 10.1056/NEJM199710023371407 PMC2851235

[obaa010-B89] Xie L, Kang H, Xu Q, Chen MJ, Liao Y, Thiyagarajan M, O’Donnell J, Christensen DJ, Nicholson C, Iliff JJ, et al 2013 Sleep drives metabolite clearance from the adult brain. Science 342:373–7. 24136970 10.1126/science.1241224 PMC3880190

[obaa010-B90] Ydenberg RC, Butler RW, Lank DB, Guglielmo CG, Lemon M, Wolf N. 2002 Trade-offs, condition dependence and stopover site selection by migrating sandpipers. J Avian Biol 33:47–55.

[obaa010-B91] Zhang SL, Yue Z, Arnold DM, Artiushin G, Sehgal A. 2018 A circadian clock in the blood-brain barrier regulates xenobiotic efflux. Cell 173:130–9.e10. 29526461 10.1016/j.cell.2018.02.017 PMC5866247

