# Supplementary material for: Energy Stores, Oxidative Balance, and Sleep in Migratory Garden Warblers (Sylvia borin) and Whitethroats (Sylvia communis) at a Spring Stopover Site
Source: Integr Org Biol. 2020 Apr 15;2(1):obaa010. doi: 10.1093/iob/obaa010 (PMC7671129; doi:10.1093/iob/obaa010)
Supplement: obaa010_Supplementary_Data [file obaa010_supplementary_data.zip › Supplementary Table 1.docx]

**Supplementary Table 1**

| Spearman's correlation test | Garden Warbler | | | | Whitethroat | | | |
| --- | --- | --- | --- | --- | --- | --- | --- | --- |
|  | Fat Score | | Muscle score | | Fat Score | | Muscle score | |
|  | r | pvalue | r | pvalue | r | pvalue | r | pvalue |
| AOX | 0,143 | 0,264 | 0,074 | 0,564 | 0,222 | 0,107 | 0,038 | 0,785 |
| dROMs | -0,021 | 0,870 | 0,135 | 0,292 | 0,040 | 0,773 | 0,200 | 0,149 |
| Oxidative Stress | -0,104 | 0,416 | 0,104 | 0,416 | 0,030 | 0,831 | 0,135 | 0,331 |
