# Supplementary material for: Energy Stores, Oxidative Balance, and Sleep in Migratory Garden Warblers (Sylvia borin) and Whitethroats (Sylvia communis) at a Spring Stopover Site
Source: Integr Org Biol. 2020 Apr 15;2(1):obaa010. doi: 10.1093/iob/obaa010 (PMC7671129; doi:10.1093/iob/obaa010)
Supplement: obaa010_Supplementary_Data [file obaa010_supplementary_data.zip › Supplementary Table 2.docx]

**Supplementary Table 2**

|  |  | Sleep Nocturnal ~ AOX | | Sleep Diurnal ~ AOX | | AOX ~ Fat score | | AOX ~ Muscle score | |
| --- | --- | --- | --- | --- | --- | --- | --- | --- | --- |
|  | AOX value | ∆ Intercept | ∆ Estimate (AOX) | ∆ Intercept | ∆ Estimate (AOX) | ∆ Intercept | ∆ Estimate (Fat score) | ∆ Intercept | ∆ Estimate (Muscle score) |
| Garden Warblers | 393,624 | -0,092 | 0,109 | 0,317 | -0,377 | 0,225 | -0,118 | 0,028 | 0,054 |
|  | 613,465 | -0,235 | 0,257 | -1,121 | 1,224 | -0,029 | 0,447 | 0,092 | 0,181 |
| Whitethroats | 470,115 | 0,310 | -0,349 | 0,717 | -0,809 | -0,139 | 0,374 | -0,010 | 0,139 |
|  |  |  |  |  |  |  |  |  |  |
|  |  | Sleep Nocturnal ~ dROMs | | Sleep Diurnal ~ dROMs | | dROMs ~ Fat score | | dROMs ~ Muscle Score | |
|  | dROMs value | ∆ Intercept | ∆ Estimate (dROMs) | ∆ Intercept | ∆ Estimate (dROMs) | ∆ Intercept | ∆ Estimate (Fat score) | ∆ Intercept | ∆ Estimate (Muscle score) |
| Garden Warblers | 20,584 | 0,075 | -0,105 | -0,418 | 0,584 | -0,012 | 0,184 | 0,038 | 0,075 |
|  | 25,475 | -0,508 | 0.666 | -0.676 | 0.886 | -0.987 | 1,348 | -0,888 | 1,094 |
| Whitethroats | 20,095 | 0,507 | -0,642 | -0,611 | 0,773 | 0,241 | 0,000 | -0,012 | 0,164 |
