# Supplementary figures and images for: Energy Stores, Oxidative Balance, and Sleep in Migratory Garden Warblers (Sylvia borin) and Whitethroats (Sylvia communis) at a Spring Stopover Site
Source: Integr Org Biol. 2020 Apr 15;2(1):obaa010. doi: 10.1093/iob/obaa010 (PMC7671129; doi:10.1093/iob/obaa010)

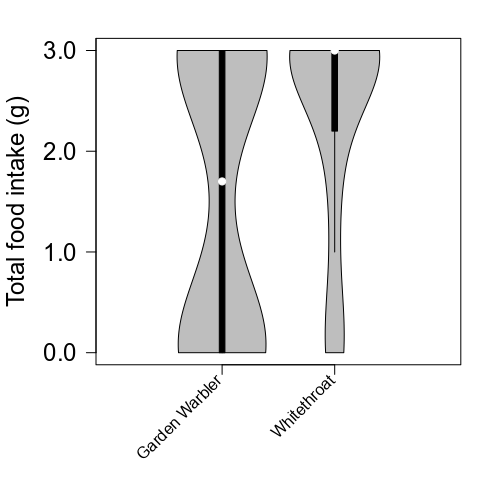

Supplement: obaa010_Supplementary_Data [file obaa010_supplementary_data.zip › SupplementaryFigure1.tiff]

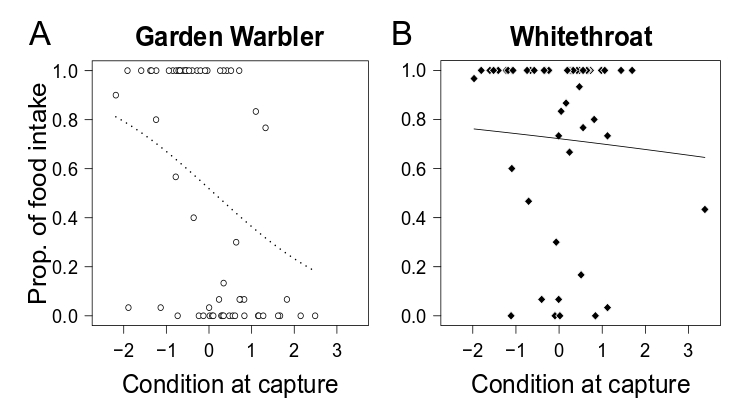

Supplement: obaa010_Supplementary_Data [file obaa010_supplementary_data.zip › SupplementaryFigure2.tiff]

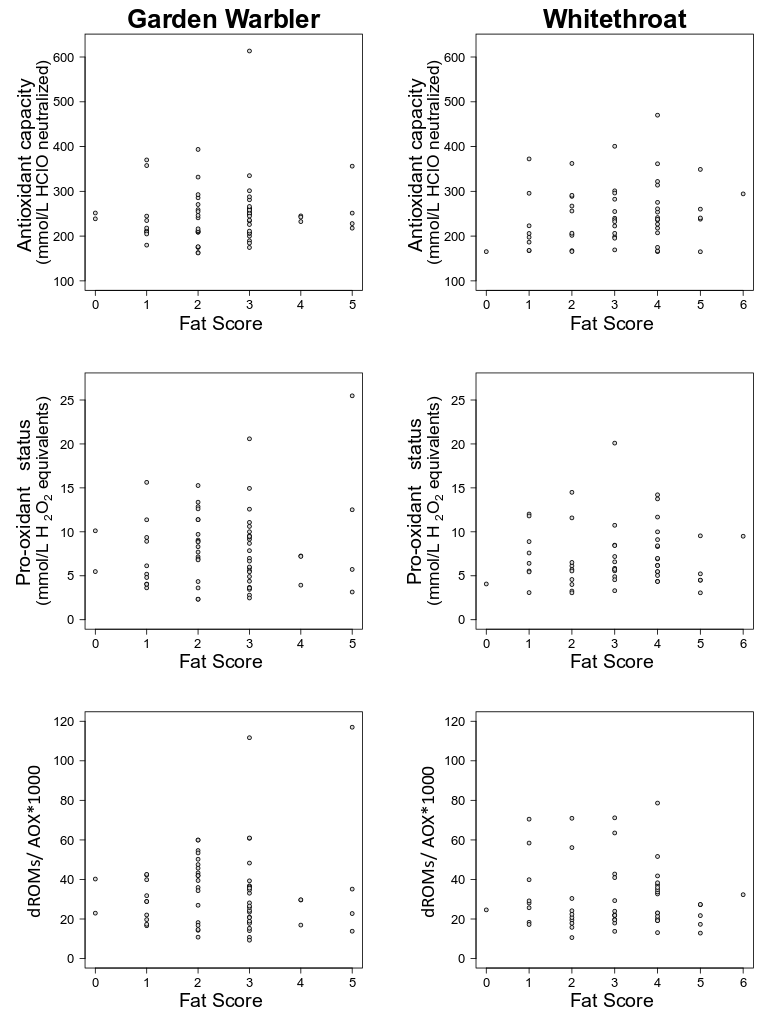

Supplement: obaa010_Supplementary_Data [file obaa010_supplementary_data.zip › SupplementaryFigure3.tiff]

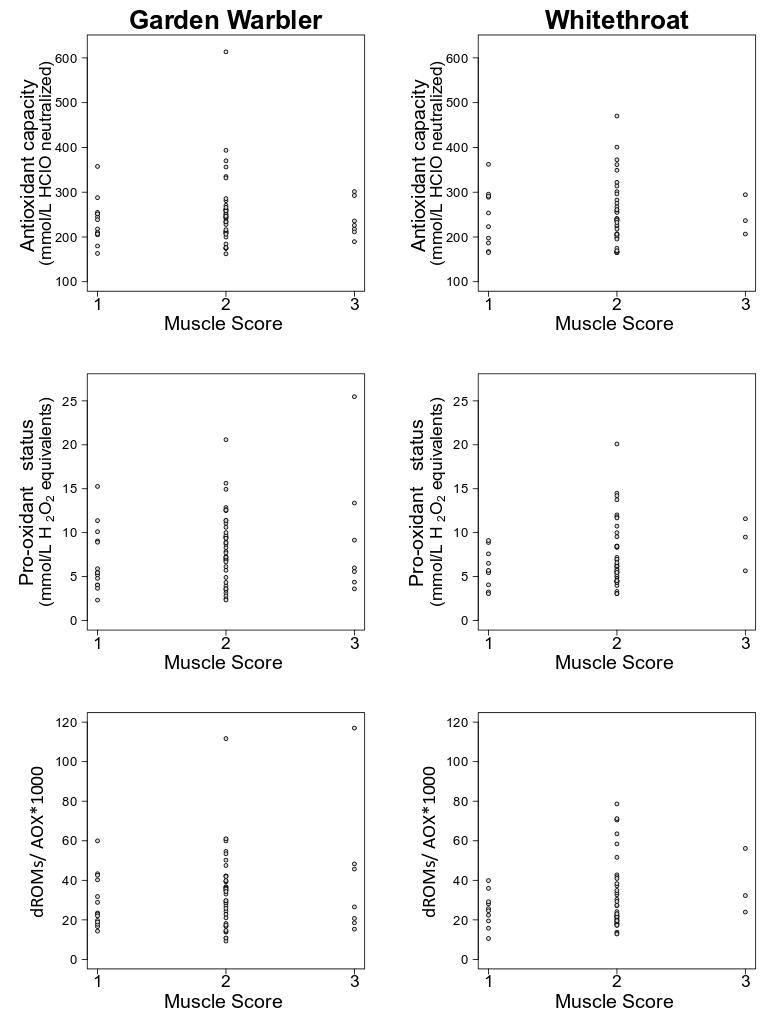

Supplement: obaa010_Supplementary_Data [file obaa010_supplementary_data.zip › SupplementaryFigure4.tiff]

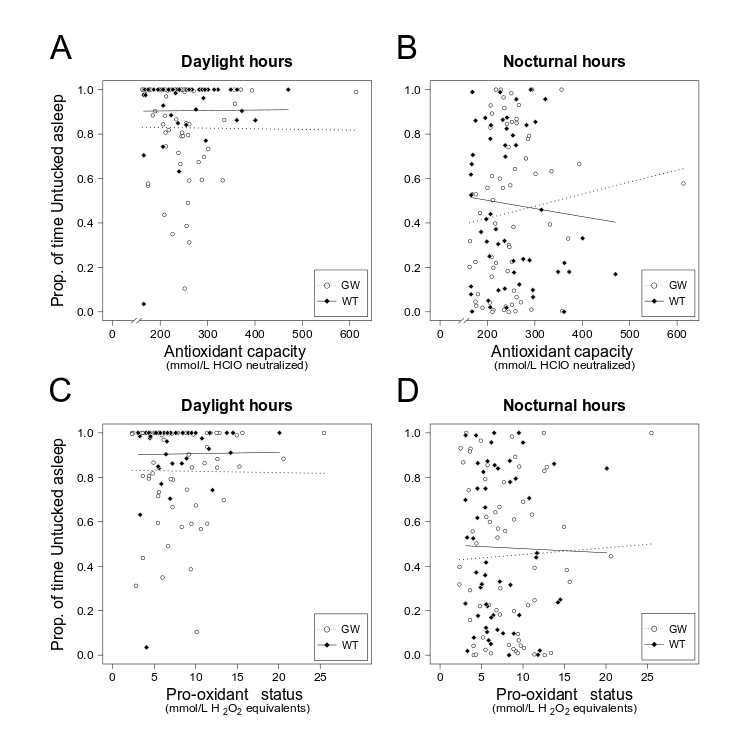

Supplement: obaa010_Supplementary_Data [file obaa010_supplementary_data.zip › SupplementaryFigure5.tiff]
